# Sex Disparity in Food Allergy: Evidence from the PubMed Database

**DOI:** 10.1155/2009/159845

**Published:** 2009-07-02

**Authors:** Caleb Kelly, Venu Gangur

**Affiliations:** ^1^Nutritional Immunology Program, Food Allergy and Immunology Laboratory, Department of Food Science and Human Nutrition, Michigan State University, East Lansing, MI 48824, USA; ^2^National Food Safety & Toxicology Center, Michigan State University, East Lansing, MI 48824, USA

## Abstract

Food allergies are potentially fatal immune-mediated disorders that are growing globally. The relationship between sex and food allergy remains incompletely understood. Here we tested the hypothesis that, should sex influence the clinical response to food allergens, this would be reflected by a sex disparity in published studies of food allergy. We performed a systematic search of the PubMed literature for IgE-mediated allergy to 11 allergenic foods of international regulatory importance. No date restriction was used and only articles in English were considered. Of the 4744 articles retrieved, 591 met the inclusion criteria representing 17528 subjects with food allergies. Whereas among children with food allergies, 64.35% were males and 35.65% were females (male/female ratio, 1.80), among adults 34.82% were males and 65.18% were females (male/female ratio, 0.53). Consequently, these data argue that there is need for further investigation to define the role of sex in the pathogenesis of food allergy.

## 1. Introduction

Immediate hypersensitivity reactions to food, commonly called food allergies, are increasing globally at an alarming rate [1]. Up to 6% of young children and 3-4% of adults in developed countries are afflicted with these potentially fatal immune-mediated disorders [2]. Since food allergies usually begin in childhood, with some food allergies such as peanut and tree nut allergies not often outgrown even after achieving adulthood, and because life-threatening reactions could be elicited from very small quantities of food proteins, these allergies are considered critical health problems. However, factors that determine clinical outcome of exposure to allergenic foods remain incompletely understood at present. Identification of such factors is crucial for the prevention and management of food-induced allergic reactions.

Although any food is capable of triggering an allergic reaction in a sensitized subject, more than 90% of food allergies are triggered by the following 11 food types: cow's milk, egg, wheat, soy, fish, shellfish, peanut, tree nuts (cashew, walnut, hazelnut, etc.), sesame, celery, and mustard seed [3–5]. Consequently, these foods are currently regarded as “red-flag” allergenic foods for regulatory purposes in the USA (first eight foods only), Canada (first nine foods only) and the European Union (all eleven foods) [3–5]. 

Food allergies are considered complex genetic disorders that are strongly influenced by the environment as evidenced by the recent rise in the food allergy prevalence around the globe [1]. Although sex has been identified as an important factor influencing the clinical presentation of asthma, the specific role of sex in food allergy remains incompletely understood [6]. A dimorphic sex distribution for asthma in which male children and female adults predominate has been reported [7]; there is also a discrepancy in sex among hospitalizations for anaphylaxis attributed to food allergy in Australia that sharply increased among children age 0–4 years during a 10 year study period [8]. However, relationship between sex and food allergy during the entire human lifetime has not been studied. Furthermore, a systematic analysis of sex-wise distribution of food allergy to all eleven allergenic foods of international regulatory importance has not been performed before.

Here we tested the hypothesis that, should sex influence the clinical response to food allergens, this would be reflected by a sex disparity in the published studies of food allergy. We performed a systematic analysis of the PubMed literature for sex versus reporting of food allergy to 11 major allergenic food types. This analysis included a substantial number of allergic individuals that allowed stratification into narrow age categories and analysis of sex distribution of IgE-mediated food allergies. We found significant evidence for sex disparity in food allergy reporting both among children and adults. These data argue that there is need for further investigation to precisely define the role of sex in the pathogenesis of food allergy.

## 2. Materials and Methods

We searched the PubMed database (on February 4, 2006) using the following search terms: “milk allergy, ” “egg allergy,” “fish allergy,” “shellfish allergy,” “mollusk allergy,” “lobster allergy,” “crayfish allergy,” “crab allergy,” “shrimp allergy,” “snail allergy,” “clam allergy,” “oyster allergy,” “nut allergy,” “pine-nut allergy,” “pinon allergy,” “pignolia allergy,” “pecan allergy,” “brazil nut allergy,” “cashew allergy,” “almond allergy,” “pistachio allergy,” “walnut allergy,” “hazelnut allergy,” “filbert allergy,” “macadamia allergy,” “chestnut allergy,” “coconut allergy,” “acorn allergy,” “peanut allergy,” “wheat allergy,” “soy allergy,” “sesame allergy,” “mustard allergy,” and “celery allergy.” No date restriction was used, and only articles in English were considered.

Search results were screened for published studies of IgE-mediated immediate hypersensitivity reactions to ingested food that included information on sex and age or classification of the subject as child (<18 years) or adult (≥18 years). Reactions were categorized as IgE-mediated if the subject reported a clinical history of food allergy and met at least one of the following criteria: an IgE reaction was specified in the publication, skin testing was positive and immediate to a food causing allergy-like symptoms after ingestion, or specific IgE antibodies were identified to a food causing allergy-like symptoms after ingestion. Participants in three national prevalence surveys [9–11] and participants of a national food allergy registry [12] were also included in our analysis although these studies included self-reported data. These studies were included because the criteria used to classify food allergy was stringent and the authors had shown previously that 93% of subjects meeting these criteria had proven IgE antibody to the implicated food. Similarly, the food allergy registry data were included because it was reported either by members of the Food Allergy and Anaphylaxis Network or by their physicians. Studies that included subjects with both IgE- (immediate) and non-IgE–mediated (delayed) reactions were excluded unless those with IgE- and non-IgE–mediated reactions could be dissociated. In several instances the literature search returned multiple publications produced with data obtained from a single group of subjects. Such subjects were included only once in the analysis. The sex distribution across age groups was analyzed using the chi-squared for trend test with GraphPad software (InStat version 3.06), and significance level was set at *P* < .05.

## 3. Results and Discussion

**Figure 1:**
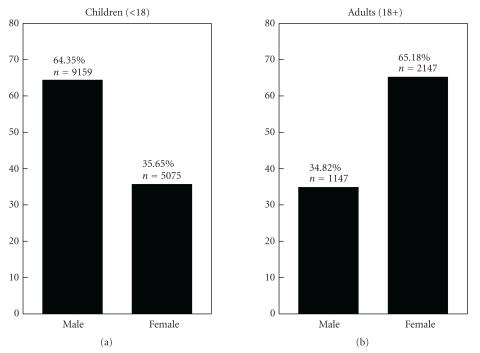
*Sex-wise distribution of food allergy in humans in the PubMed database*. All reported subjects allergic to one or more of 11 major allergenic foods were divided into two groups of <18 and ≥18 years of age.

**Figure 2:**
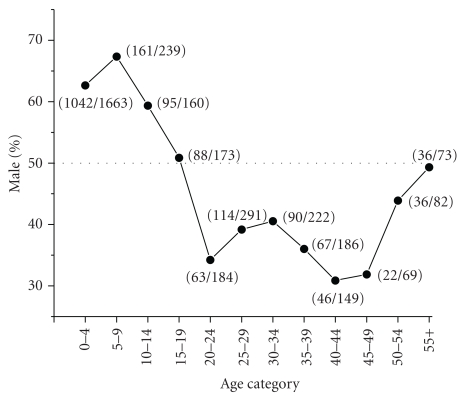
*Sex disparity in food allergy over the human life-span*. These data show the percent males with food allergy over the human life-span. There is disproportionate representation of males during early life and females during middle age among humans in the food allergy literature accessed with the PubMed database. A notable cross-over in the sex ratio occurs during the teen years and the sex disparity is diminished in age categories after 50 years.

Our search of the PubMed database returned 4744 articles. Of these, 591 met the inclusion criteria, representing 17,528 food-allergic individuals. Analysis by age and sex revealed a sex bias in food allergy reporting (Figure 1). Among the food allergic children (<18 years age) included in the analysis, 9159 (64.35%) were male and 5075 (35.65%) were female (male/female ratio of 1.80). Of the adults (≥18 years age) 1147 (34.82%) were male and 2147 (65.18%) were female (male/female ratio of 0.53). When information on specific ages was available, the data were further analyzed by classifying subjects into subgroups of age with five-year increments (Figure 2). This analysis once again revealed a sex disparity in food allergy reporting, in which males predominated at 0–4, 5–9, and 10–14 years age groups. No difference was apparent in the sex distribution in the 15–19 years age group. In older age groups most food allergy reporting was among females, until the age of 50–54 years, at which point the food allergy reporting was similar in both sexes (chi-squared for trend, *P* < .001). Age at onset of food allergy was used in the analysis when information was available. The reporting of childhood onset of food allergy was similar in males (5.84% among males) and females (5.12% among females).

This is the first systematic analysis of the PubMed literature examining the relationship between sex and reporting of food allergy to all the major 11 allergenic foods of international regulatory importance. In addition, this is also the first study examining the relationship between sex and food allergy reporting over the entire human lifespan. Our data provide evidence for the existence of sex disparity in published studies of food allergy in the PubMed database.

The dimorphic distribution of food allergy reporting (i.e., most reports of food allergies among children involves boys and most reports of food allergies among adults involves women) that we found shows striking similarity to that of asthma reported earlier; this may reflect a general pattern among atopic diseases [7]. However in comparison to asthma studies, little descriptive detail, such as age-wise distribution, was available in food allergy studies. Poulos et al. described the sex distribution of individuals hospitalized for food-related anaphylaxis in Australia from 1994/1995 to 2004/2005 [8]. They reported anaphylaxis attributed to food in four age categories, two of which can be directly compared with our analysis. In the 5- to 14-year-old age group, Poulos et al. reported a male/female ratio of 1.54 (95% CI, 1.30–1.81), compared to a ratio of 1.79 in our analysis of the same age group. In the 15- to 34-year-old age group, they reported a male/female ratio of 0.72 (95% CI, 0.63–0.88) compared to 0.69 in our analysis of the same ages. Similar to our analysis, Poulos et al., found that women predominate in middle age (35- to 64-year-old age group) and no difference was observed in their oldest age category (≥65 years old). Notably, the nature of our study allowed us to include a greater number of subjects for analysis and enabled us to categorize the data into 5-year increments. This permitted greater precision in describing the sex distribution over the human lifespan.

Our analysis is also corroborated by the findings of a random telephone survey conducted by Sicherer et al. in 2003 that was designed to estimate the prevalence of peanut and tree nut allergies in the United States [10]. Their survey, representing 4855 households, found 26 allergic male and 10 allergic female children (<18 years) and 37 allergic male and 82 allergic female adults (≥18 years). Another survey by the same group was conducted in 2004 to determine the prevalence of seafood allergies [11]. This survey represented 5529 households in the United States and reported 15 allergic male and 8 allergic female children (<18 years) and 99 allergic male and 207 allergic female adults (>18 years). Our analysis included reports not only of the above foods, but also eight additional major allergenic foods of international regulatory significance. Therefore, the pattern of sex disparity in food allergy reporting that we report is independent of the specific type of the allergenic food.

The appearance of sex reversal in food allergies at puberty (females, 10–14 years; males, 12–16 years) along with the attenuation of sex difference around the age of menopause (45–55 years) suggests a role for sex hormones in food allergies. This theory is supported by observations that the female immune response changes throughout the menstrual cycle. One study examining skin prick testing (SPT) in women with aeroallergens reported significantly increased wheel-and-flare responses on days 12–16 of the menstrual cycle which correspond to peak estrogen levels [13]. Kirmaz, et al. compared allergen SPT with serum hormone levels in 42 women with seasonal allergies. They found that estradiol and luteinizing hormone were correlated with SPT response at mid-cycle [14]. The menstrual phase has also been shown to influence nasal reactivity, as the period of peak estrogen is correlated with the nasal mucosa becoming hyperreactive to histamine [15].

It is well known that sex influences immune responses. Thus, women exhibit a more robust antibody response to infection and vaccination than do men [16]. Similarly, in mouse model studies, intranasal sensitization of CBA/J mice to phospholipase A2 resulted in significantly more specific IgE in females than males [17]. Furthermore, castration of male mice resulted in a significant increase of specific IgE which was reversed with testosterone treatment.

Estrogens and androgens appear to have opposing effects on humoral immunity. Testosterone has been shown to reduce immunoglobulin production by inhibiting differentiation and promoting apoptosis of immature B cells [18, 19] whereas estrogens enhance humoral immunity by stimulating B cell differentiation and immunoglobulin production [20, 21]. However the specific impact of estrogens compared with androgens on IgE response to food allergens is not known. Other cells of the innate and specific immune system, such as T cells, monocytes, mast cells, and neutrophils express receptors for sex hormones [16, 22]. Generally speaking, androgens promote Th1 polarization characterized by IL-2 production [22] while estrogens and progesterone promote Th2 polarization characterized by the production of IL-4, IL-5, IL-10, and IL-13 [16, 22]. Future studies investigating the effect of sex on food allergy relevant immune function might consider evaluating not only the impact of individual sex hormones but also the relative ratio of androgen to estrogen.

The immune-modulating effect of sex hormones provides a convenient explanation of the sex distribution that we found in food allergies. However, the impact of sex may be complex and other hypotheses might explain the observation. For example, a sex-based difference in response to metabolic factors may influence immune function. Among sexually mature females, a high body mass index is associated with atopy [23], allergy [24], and asthma [25]. This might indicate a role for leptin or other adipokines in susceptibility to allergic disease [26]. Although leptin promotes a Type 1 immune response, it is positively associated with IgE and asthma in boys [27]. In adults, Sood et al. reported a positive association between leptin and asthma that is stronger in adult females than males [28]. In one study, administration of leptin to animals at levels corresponding with modest obesity resulted in higher serum IgE, indicating a possible direct link between leptin and allergic disease [29]. Notably, the relationship between obesity or leptin and allergic response to food proteins is not clear and is likely to be complex because obesity is associated with increased serum estrogen concentrations [30] and leptin itself may promote the production of estrogen and the activation of estrogen receptors [31].

The hormone hypothesis more aptly explains the sex distribution of food allergy after puberty, when sex hormone production increases, than the difference that we found during childhood. Genetic predisposition could explain the increased reporting of food allergy among prepubescent-aged boys in our analysis. The sexual genotype (XX in females and XY in males) is the basis of sex differences, and an X-linked recessive trait associated with allergic disease would be more likely to be unmasked in males and could explain the predominance of food allergies in males at a very young age. This pattern of genetic inheritance has been suggested to explain the higher prevalence of male wheezing in a study of Swedish infants [32]. Higher levels of total IgE is present in the cord blood of males compared to females [33] and remains higher throughout the lifecycle despite the female tendency toward atopy after puberty [34]. One factor that may be responsible for the difference is a polymorphism in the CTLA4 gene that is correlated with cord blood IgE in males [35]. Further study of such polymorphisms may lead to early identification and targeted intervention for those at high risk for atopic diseases.

Our method of literature analysis to describe the sex distribution in food allergy provided a large number of allergic individuals and thus greater descriptive detail. However, this analysis inherits any selection bias present in the primary literature. For example, sex and gender differences including the likelihood to seek treatment, willingness to participate in research studies, perception of symptoms, and so forth, may be reflected in our results. Of particular interest is the greater tendency for women to report symptoms of food allergy [6]. Unfortunately the exact contribution this may have had toward our results cannot be determined and is an important topic for future research. Yet this is not likely to undermine the pattern of distribution because a markedly similar pattern has been described for epinephrine dispensing [36] and all cause anaphylaxis [37] and hospitalization for food-related anaphylaxis [8]. Therefore, it is important to identify the factor(s) responsible for the sex disparity of food allergy as well as other atopic diseases. It is plausible that the distribution of food allergy in adulthood is influenced by cross reactivity of food allergens with aeroallergens in individuals sensitized to pollen (i.e., oral allergy syndrome). In this scenario, sex differences in food allergy are driven by disproportionate rates of respiratory sensitization to non-food antigens rather than primary sensitization to food. Variation in methods for determining positive skin-prick and food-specific IgE exist between studies. This methodological heterogeneity makes evaluation in future large scale studies important in confirmation of the sex-age distribution in food allergy. In addition, confirmation of our findings by metaanalysis is important because this would permit accounting for the sample size of each study individually. Since maternal oral contraceptive use may increase the risk of allergic disease among offspring [38], it should be determined whether contraception alters the risk of food allergy. The effects of pregnancy and parity as well as those of dietary phytoestrogen consumption and exposure to environmental xenoestrogens may also be important [39]. Understanding the genetic basis of food allergy with a focus on sex-specific modification of immune response to allergenic foods may eventually lead to targeted interventions and early identification of those at greatest risk.

Appropriate use of the terms “sex” and “gender” are important. Sex refers to biologic differences between male and female while gender refers to social, cultural, and/or psychological traits typically associated with one sex [40]. In this analysis of the food allergy literature we emphasize biologic explanations and often utilize the term “sex.” Factors related to gender may also contribute to the unequal reporting of food allergy. In response to the dearth of research on the interaction between gender and food allergy DunnGalvin et al. have highlighted the need to incorporate a gender dimension in food allergy research [6].

The U.S Food Allergen Labeling and Consumer Protection Act specifies “Crustacean shellfish” and thus excludes other forms of shellfish such as mollusks. The Canadian Food Inspection Agency is broader in their classification of seafood allergens. They include “Fish, Crustaceans and Shellfish” and they clarify the difference between crustaceans and shellfish categories (under which mollusks are categorized) [41]. In this study, the literature search was designed to be inclusive of foods commonly causing allergies as recognized by government agencies in the United States as well as the European Union and Canada.

## 4. Conclusions

Our analyses provide what we believe to be the first evidence that there exist an unequal reporting of food allergy to11 major allergenic foods between males and females. Whereas among children, most studies involve males, among adults, most studies involve females. Thus, there is a dimorphic sex distribution in the food allergy literature. This finding reinforces the importance of including sex and gender as important factors in all investigations—clinical as well as translational food allergy research.

Sex can theoretically impact the pathogenesis of food allergy at one or more stages of allergy disease development, from the exposure to allergenic foods, immune system processing, and presentation of allergenic epitopes to an IgE response leading to sensitization and clinical expression of the disease upon re-exposure. In Table 1, we summarize a number of research questions that our findings have evinced. Future work might focus on addressing these questions not only in human subjects with food allergy but also in appropriate animal models of food allergy.

In conclusion, there is evidence in the PubMed database for a sex disparity in published studies of food allergy. Consequently, these data argue that there is need for further investigation to precisely define the role of sex and gender in the pathogenesis of food allergy.

## Figures and Tables

**Table 1:** Sex disparity in food allergy reporting: implications for clinical and translational research.

Findings from our research	Questions to consider
*In children*:	- Does maternal imprinting and/or epigenetic modification differentially target sex *in-utero* with regard to food allergy?
*Greater than 64% of the reported food allergies to 11 major foods involve boys*	- Is there a difference in exposure to allergenic foods among males and females?
	- Is there a sex disparity in neonatal/postnatal immune response to allergenic foods?
	- Is there a sex disparity in the quality (intensity and/or frequency) of clinical response to allergenic foods upon re-exposure among children?

*In adults*:	- Is there a sex disparity in the outgrowing of food allergy during late childhood or adulthood?
*Greater than 65% of the reported food allergies to 11 major foods involve women*	- Is there a difference in exposure to allergenic foods among males and females?
	- What is the impact of the menstrual cycle, pregnancy, and sex hormones on allergic response to foods?
	- Is there a sex disparity in the quality (intensity and/or frequency) of clinical response to allergenic foods upon re-exposure?
	- Do psychosocial factors contribute to sex disparity in food allergy reporting?
	- Does the sex disparity reflect a difference in food allergy or sensitization to aeroallergens resulting in oral allergy syndrome?
